# Social cognition impairments are associated with behavioural changes in the long term after stroke

**DOI:** 10.1371/journal.pone.0213725

**Published:** 2019-03-15

**Authors:** Britta Nijsse, Jacoba M. Spikman, Johanna M. A. Visser-Meily, Paul L. M. de Kort, Caroline M. van Heugten

**Affiliations:** 1 Elisabeth-Tweesteden Hospital, Department of Neurology, Tilburg, The Netherlands; 2 University of Groningen, Department of Clinical and Developmental Neuropsychology, Groningen, The Netherlands; 3 University Medical Center Groningen, Department of Neurology, Groningen, The Netherlands; 4 Center of Excellence in Rehabilitation Medicine, collaboration between University Medical Center Utrecht and Hoogstraat Rehabilitation, Utrecht, The Netherlands; 5 Brain Center Rudolf Magnus, University Medical Center Utrecht, Department of Rehabilitation, Physical Therapy Science and Sports, Utrecht, The Netherlands; 6 Maastricht University, Faculty of Psychology and Neuroscience, Department of Neuropsychology and Psychopharmacology, Maastricht, The Netherlands; 7 Maastricht University Medical Center, Faculty of Health, Medicine and Life Sciences, School for Mental Health and Neuroscience, Maastricht, The Netherlands; Univdersity Hospital of TübingenUniversitatsklinikum Tubingen, GERMANY

## Abstract

**Background and purpose:**

Behavioural changes after stroke might be explained by social cognition impairments. The aim of the present study was to investigate whether performances on social cognition tests (including emotion recognition, Theory of Mind (ToM), empathy and behaviour regulation) were associated with behavioural deficits (as measured by proxy ratings) in a group of patients with relatively mild stroke.

**Methods:**

Prospective cohort study in which 119 patients underwent neuropsychological assessment with tests for social cognition (emotion recognition, ToM, empathy, and behaviour regulation) 3–4 years post stroke. Test scores were compared with scores of 50 healthy controls. Behavioural problems were assessed with the Dysexecutive Questionnaire (DEX) self rating and proxy rating scales. Pearson correlations were used to determine the relationship between the social cognition measures and DEX scores.

**Results:**

Patients performed significantly worse on emotion recognition, ToM and behaviour regulation tests than controls. Mean DEX-self score did not differ significantly from the mean DEX-proxy score. DEX-proxy ratings correlated with tests for emotion recognition, empathy, and behavioural regulation (lower scores on these items were associated with more problems on the DEX-proxy scale).

**Conclusions:**

Social cognition impairments are present in the long term after stroke, even in a group of mildly affected stroke patients. Most of these impairments also turned out to be associated with a broad range of behavioural problems as rated by proxies of the patients. This strengthens the proposal that social cognition impairments are part of the underlying mechanism of behavioural change. Since tests for social cognition can be administered in an early stage, this would allow for timely identification of patients at risk for behavioural problems in the long term.

## Introduction

Behavioural changes are a frequent complication after stroke and may have a negative impact on the quality of life of patients, but also on the quality of life of caregivers.[1] Since behavioural changes often relate to inadequate or inappropriate social-emotional behaviour, for example hurtful or insulting communication and emotional indifference, it is plausible to assume that social cognition impairments are part of the underlying mechanism of behavioural change.[2] Social cognition comprises the capacities of individuals to process social information, that is, to understand the behaviour of others and to react adequately in social situations. These capacities involve different, but interrelated, processes.[3,4] First, it requires the ability to recognize other people’s emotions, e.g. by facial expressions. Second, intentions, dispositions and beliefs of others have to be inferred by forming a Theory of Mind (ToM). Furthermore, one should be able to empathize with others by linking other people’s emotions to one’s own emotional experience.[3] A final element is behaviour regulation, which involves monitoring, control and inhibition of one’s own behaviour, emotions, or thoughts, in accordance with the demands of the situation.[5] Collectively, all these skills facilitate appropriate social behaviour. And consequently, impairments in social cognition might be related to disturbances in social-interpersonal behaviour. Social cognition impairments have been found in stroke patients, with evidence for deficits in emotion recognition[6], ToM[7–9] and empathy[10]. To date, the relation between these impairments and social-behavioural problems has not been investigated yet.

Since behavioural changes are commonly perceived by caregivers, they should not only be explored by patient-, but also by proxy reports. We learned from earlier research that the patients’ and relatives’ views on behavioural disturbances after stroke may differ substantially, with, in general, patients reporting less problems than relatives.[1,11] This disagreement may result from patients’ impaired self-awareness, but denial may also be involved.[11] Although denial may also play a role in relatives, their ratings of patients’ behaviour are generally considered more objective and accurate. Therefore, proxy reports are essential in assessing behavioural changes.

In patients with traumatic brain injury (TBI) proxy ratings have been used to examine the social-behavioural consequences of social cognition impairments.[12–14] Spikman et al[14] concluded that poor emotion recognition was associated with behavioural problems in TBI-patients, as rated by proxies. To our knowledge, there are no studies that investigated the behavioural consequences of social cognition impairments in stroke patients. However, according to caregivers the most frequent residual symptom identified as among the top five most important problems in stroke patients, was impaired recognition of the emotions of others (loss of emotional empathy), followed by ‘change in personality and behaviour’.[15] This indicates the relevance of social cognitive and behavioural consequences of stroke.

The aim of the present study was therefore to investigate whether performances on social cognition tests (including emotion recognition, ToM, empathy and behaviour regulation) were associated with behavioural deficits in stroke patients, as measured by proxy ratings. Furthermore, self-awareness of stroke patients, and its association with social cognition deficits, was explored.

## Methods

### Design

The current study is an extension of the prospective longitudinal multicentre Restore4Stroke cohort study, in which stroke patients and their caregivers were followed for two years including five measurements (T1-T5).[16] Patients were recruited from stroke units in six participating hospitals in the Netherlands between March 2011 and March 2013. For the present study patients and caregivers were asked to participate in an extra assessment at 3–4 years post stroke (T6). The T6 measurements were conducted between July 2015 and October 2016. The Restore4Stroke cohort study and the extra follow-up measurements reported here were approved by the Medical research Ethics Committees United (MEC-U).

### Subjects

Patients were eligible for this study if they had a clinically confirmed diagnosis of stroke (ischemic or hemorrhagic, judged from computed tomography scan in the acute phase, according to the standard care in the participating hospitals). This is in line with the current American Stroke guidelines, which state that the diagnosis of stroke is a clinical diagnosis.[17] Whenever the clinician was in doubt about a patient’s symptoms, magnetic resonance imaging (MRI) of the brain was performed to establish the diagnosis. All patients had to be at least 18 years old. Patients were excluded if they (1) had a serious other condition whereby an interference with the study outcomes was expected (e.g. neuromuscular disease); (2) were already dependent regarding activities of daily living (ADL) before their stroke as defined by a Barthel Index (BI) of ≤17; (3) had insufficient command of the Dutch language to understand and complete the questionnaires; or (4) were already suffering from cognitive decline as defined by a score of ≥1 on the Heteroanamnesis List Cognition[18] before their stroke. Patients with evidence of visual neglect or a language disorder were excluded as well, because their results on the social cognition tests might have been influenced by this.

Proxies of the patients (partners, family members, friends or acquaintances) were contacted by the research assistant to fill out the Dysexecutive Questionnaire (DEX) proxy version.

Healthy controls for social cognition testing were recruited in two ways. First, partners of the participating stroke patients were asked to act as healthy controls. Second, data from an additional control group, who took part in another study, were added. These controls had been recruited from acquaintances of the researchers. Exclusion criteria were the same as for patients, with an additional exclusion criterion of the occurrence of transient ischemic attack (TIA) or stroke. Informed consent was obtained from all patients, proxies and healthy controls.

### Procedure

Three-four years after stroke (T6) an extensive neuropsychological assessment was conducted by a trained research assistant (graduate neuropsychologist), either in the nearest participating hospital or at home (if patients were not able to travel). Patients performed the total neuropsychological test battery, controls only performed the social cognition test battery.

Patients and proxies respectively filled out the DEX-self and DEX-proxy rating scale.

### Measures

Demographic characteristics included sex, age and level of education. Patients’ level of education was recorded according to a Dutch classification system ranging from 1: did not finish primary school, to 7: university education.[19]

The hemisphere involved, the type of stroke (ischemic or hemorrhagic) and history of previous stroke(s) were obtained from medical charts. Severity of stroke was assessed with the National Institutes of Health Stroke Scale (NIHSS).[20] ADL was assessed with the BI.[21] The Bells test was used for the evaluation of visual neglect[22], and the Boston Naming Test (BNT)[23] for the evaluation of language (naming) disorder.

The Dysexecutive Questionnaire (DEX)[24] is a 20-item questionnaire, designed to measure behavioural changes that can be part of the dysexecutive syndrome. The score ranges from 0–80 (0–4 per item), with higher scores representing more severe behavioural problems. The DEX has a self rating (DEX-self) and proxy rating (DEX-proxy) version. The individual DEX items are displayed in Table 1. Since it is a broad measure, Simblett and Bateman[25], and Bodenburg and Dopslaff[26] performed factor and Rasch analyses to unravel the structure of the DEX resulting in a division into different subscales, representing different aspects of behavioural changes. Simblett and Bateman made a division into three subscales: Executive Cognition scale (DEX-EC: items 1,4, 6 and 18)[25], which measures executive functioning (planning, regulation, focussing and switching); Metacognition scale (DEX-MC: items 2, 5, 12, 16 and 20)[25], which measures awareness and understanding of one’s own thought processes; and Behavioural-Emotional Selfregulation scale (DEX-BESR: items 3, 7, 8, 10, 13, 14, 15 and 17)[25], which measures functions that are involved in emotional and reward processing, necessary for appropriate adaptive responding to others. Bateman and Dopslaff defined a fourth subscale: the Social Convention scale (DEX-SC: items 9, 12, 13 and 20)[26], which measures awareness of social conventions and the ability to incorporate social interaction in one’s own behaviour. The DEX-BESR and DEX-SC subscales represent measures of social-emotional processes in behaviour, while DEX-EC and DEX-MC scales are more rational scales, measuring executive functioning and reflection on one’s own behaviour respectively. The four subscales were used in our analyses.

**Table 1:** Percentage of patients and proxies reporting complaints about the patient on the separate DEX-items.

DEX-items	% Patients (n = 119)	% Proxies (n = 119)
1. Problems with abstract thinking	73.1	72.3
2. Impulsivity, acting without thinking	70.6	63.9
3. Confabulation	13.4	24.4
4. Planning problems	60.5	58.0
5. Euphoria, excitability	69.7	68.1
6. Temporal sequencing problems	61.3	58.8
7. Lack of insight and social awareness	51.3	47.1
8. Apathy and lack of drive	72.3	65.5
9. Disinhibition, inappropriate behaviour	36.1	45.4
10. Variable motivation	60.5	47.1
11. Shallow affect	72.3	61.3
12. Losing temper, aggression	73.9	76.5
13. Lack of concern	45.4	49.6
14. Perseveration	55.5	51.3
15. Restlessness	66.4	53.8
16. Inability to inhibit responses	59.7	58.0
17. Knowing-doing dissociation	53.8	45.4
18. Distractibility	80.7	73.1
19. Loss of decision making ability	73.9	63.9
20. Unconcern for social rules	68.9	64.7

DEX = Dysexecutive Questionnaire.

To measure social cognition, tests were chosen that were designed to measure emotion recognition, ToM, empathy, and behaviour regulation.

#### Emotion recognition

The Ekman 60-Faces test of the Facial Expression of Emotion: Stimuli and Tests (FEEST)[27] was used to examine the recognition of emotional expressions on faces. Sixty faces were shown, with expressions depicting the primary emotions Fear, Disgust, Anger, Happiness, Sadness, or Surprise (maximum score per emotion = 10). Stimuli were presented for 3 seconds. The total score ranges from 0–60, with higher scores indicating better emotion recognition.

#### Theory of mind

The Cartoon test[7] is a test for ToM. Subjects had to describe 12 cartoons displaying humorous situations. In half of them, the joke is based on the false belief or ignorance of a character in the cartoon, and the subject needs to form a ToM in order to understand the joke. The other cartoons only require mental state attribution of the person who drew the cartoon in order to understand his humorous intention. The score ranges from 0–36 (0–3 per item), with a higher score denoting better performance. With a short version of the Faux Pas test[28] the capacity to judge the inappropriateness of behaviour in social situations was assessed. A faux pas occurs when someone says something awkward, hurtful, or insulting to another person, not realizing that one should not say it. Recognizing a faux pas requires belief attribution and inferences about a person’s feelings. The task consists of 10 short stories, half of which describing a situation comprising a social faux pas. The Faux Pas Detection score ranges from 0–10 (higher score indicating better detection).

#### Empathy

In the five faux pas items of the Faux Pas test participants are asked to describe the feelings of the faux pas victim. These responses form the Faux Pas Empathy score, ranging from 0–5, with a higher score indicating greater empathic ability. Various aspects of emotional empathy were assessed using the Dutch version of the Balanced Emotional Empathy Scale (BEES) [29]. This is a 30-item questionnaire, on which subjects rate the extent to which they agree with each statement (ranging from—4 to 4), for example: “Unhappy movie endings haunt me for hours” or “I cannot feel much sorrow for those who are responsible for their own misery” (total score ranging from -120 to 120). Higher scores represent higher levels of emotional empathy.

#### Behaviour regulation and inhibition

The Hayling Sentence Completion test[30] consists of two sets of 15 sentences each having the last word missing. In the first section the examiner reads each sentence aloud and the participant has to simply complete the sentences, yielding a simple measure of response initiation speed. The second part requires subjects to complete a sentence with a nonsense ending word (and suppress a sensible one), giving measures of response suppression ability and thinking time. Total scaled score ranges from 1 (impaired) to 10 (very superior).

### Statistical analyses

Descriptive statistics were used to describe patients’ characteristics. Chi-square and t-tests were used to compare demographic characteristics between patients and controls. Each DEX-item was dichotomized, with a complaint being absent or present. Descriptive statistics were used to calculate frequencies of the complaints of patients and proxies.

Preliminary analyses were conducted to ensure no violation of the assumption of normality on all social cognition tests and DEX-scores.

Analysis of covariance (ANCOVA) was used to explore differences between patients and controls on all social cognition tests, in which the selection of covariates was based on demographic differences between groups. Effect sizes (Cohen’s d) were calculated using means and standard deviations. T-tests were used to compare test results between vertebrobasilar and anterior circulation patients, and to compare left- versus right-hemisphere patients.

T-tests were used to compare mean DEX-self scores and DEX-proxy scores. DEX difference scores were calculated (DEX-dif = DEX-self minus DEX-proxy) as an indication of self-awareness. Pearson correlations were used to determine the relationship between the social cognition measures and the DEX-self, DEX-proxy and DEX-dif scores, and the four DEX subscales (calculated from the DEX-proxy score).

The critical value of alpha was set at 0.05. Analyses were performed with IBM SPSS Statistics version 19.

## Results

A total of 395 patients were included in the Restore4Stroke cohort study. At T6, 160 of them (40.5%) were eligible for further testing. With respect to the 235 resigned patients, 33 patients died, 120 patients refused further participation, 47 patients could not be reached by T6, and in 35 patients it was not possible to conduct the T6 assessment because of their general physical condition. Two patients had evidence of visual neglect according to the results of the Bells test, ten patients had evidence of language disorder according to the results of the BNT or the clinical judgement of the neuropsychologist. They were all excluded, which resulted in a total of 148 patients. In 119 patients, both DEX self reports and DEX proxy reports were available, so they were included in the present study.

The demographic and stroke-related characteristics of these 119 patients are displayed in Table 2. At T6 mean age was 67.9 years (SD10.8), and mean time since stroke was 3.7 years (SD0.7).

**Table 2:** Characteristics of stroke patients (n = 119) and controls (n = 50).

	patients, n (%)	controls, n (%)
Sex, number of men	84 (70.6%)	24 (48.0%)
Age in years; mean (SD)		
T1	64.3 (±11.0)	
T6	67.9 (±10.8)	65.2 (±8.1)
Education level		
Low (1–5)	86 (72.3%	30 (60.0%)
High (6–7)	33 (27.7%)	20 (40.0%)
*Stroke characteristics*		
Type of stroke		
Ischemic	111 (93.3%)	
Haemorrhagic	8 (6.7%)	
Location of stroke		
Left anterior circulation	38 (31.9%)	
Right anterior circulation	50 (42.0%)	
Vertebrobasilar	31 (26.1%)	
Recurrent stroke	17 (14.3%)	
NIHSS score at T1; median (SD)	2.0 (±3.1)	
No stroke symptoms (NIHSS 0)	31 (26.1%)	
Minor stroke symptoms (NIHSS 1–4)	65 (54.6%)	
Moderate stroke symptoms (NIHSS 5–12)	21 (17.6%)	
Moderate to severe symptoms (NIHSS>12)	2 (1.7%)	
Barthel Index at T1		
ADL independent (BI 19–20)	68 (57.1%)	
ADL dependent (BI<19)	51 (42.9%)	

T1 = 4 days after stroke; NIHSS = National Institutes of Health Stroke Scale.

Fifty controls (half of which were partners) with a mean age of 65.2 years (SD8.1) were included. Chi-square and t-tests showed no significant differences between patients and controls with respect to age (t = -1.8, p = 0.077) and education level (high-education: controls 40.0% vs patients 27.7%, X^2^ = 2.5, p = 0.117), while there were more men in the patient group (patients 70.6% vs controls 48.0%, X^2^ = 7.8, p = 0.005). Therefore, sex was included as covariate in the ANCOVA.

In Table 3 the means and SDs on the social cognition tests are presented for both the patients and the control group. Patients performed significantly worse on the FEEST total score and FEEST emotion Anger (emotion recognition), Cartoon test (ToM), and Hayling (behaviour regulation).

**Table 3:** Social cognition test results 3–4 years after stroke.

Test measures	Stroke patients (n = 119) Mean (SD)	Healthy controls (n = 50) Mean (SD)	ANCOVA		
			F	p-value	Effect size
FEEST total score	42.66 (6.2)	45.02 (6.2)	4.26	**0.041**	0.38
FEEST-anger	6.69 (2.2)	7.79 (1.9)	7.98	**0.005**	0.54
FEEST-disgust	6.69 (2.4)	7.27 (2.1)	0.90	0.346	0.26
FEEST-fear	4.70 (2.2)	4.81 (2.5)	0.55	0.460	0.05
FEEST-happiness	9.73 (0.6)	9.79 (0.5)	0.071	0.790	0.11
FEEST-sadness	6.06 (2.0)	6.50 (1.8)	1.58	0.210	0.23
FEEST-surprise	8.78 (1.5)	8.85 (1.2)	0.069	0.793	0.05
Cartoon test	21.04 (6.9)	22.75 (5.9)	5.59	**0.019**	0.27
Faux Pas detection	9.21 (1.0)	9.12 (0.7)	0.34	0.563	-0.10
Faux Pas empathy	2.99 (1.2)	3.28 (1.2)	1.05	0.306	0.24
Hayling	3.02 (1.8)	4.65 (1.4)	32.32	**<0.001**	1.01
BEES	32.22 (21.5)	35.60 (26.1)	0.035	0.852	0.14

FEEST = Facial Expression of Emotion: Stimuli and Tests; BEES = Balanced Emotional Empathy Scale.

Table 4 shows the mean scores in the different subgroups (vertebrobasilar versus anterior circulation, and left- versus right-hemisphere). No significant differences were found between subgroups.

**Table 4:** Comparisons of mean scores between subgroups.

	Anterior circulation (n = 86)	Vertebrobasilar circulation (n = 30)	p-value
FEEST	42.7	42.5	0.893
Cartoons	20.9	21.4	0.762
Faux Pas detection	9.2	9.3	0.464
Faux Pas empathy	3.0	2.9	0.494
Hayling	3.0	3.1	0.686
BEES	32.9	30.3	0.573
	Left hemisphere (n = 36)	Right hemisphere (n = 50)	p-value
FEEST	42.3	43.0	0.639
Cartoons	21.4	20.6	0.612
Faux Pas detection	9.1	9.2	0.732
Faux Pas empathy	2.9	3.1	0.399
Hayling	2.9	3.0	0.890
BEES	36.2	30.6	0.263

FEEST = Facial Expression of Emotion: Stimuli and Tests; BEES = Balanced Emotional Empathy Scale.

Mean DEX-self score was 20.1 (SD11.5), and mean DEX-proxy score was 19.3 (SD13.4); t = 0.672, p = 0.503. On the DEX subscale items there was a significant difference between the DEX-EC self score and proxy score (mean self score = 5.0 (SD3.4) vs mean proxy score = 4.3 (SD 3.2); t = 2.1, p = 0.042). On the other 3 subscales there were no significant differences.

In Table 1 the separate DEX-items are listed along with the frequencies of the complaints of patients and proxies. The behavioural problems most frequently mentioned by patients were 1) distractibility, 2) loss of decision making ability, and 3) aggression. The behavioural problems most frequently mentioned by proxies were 1) aggression, 2) distractibility, and 3) problems with abstract thinking. On most items there were more patients than proxies reporting problems.

Correlations between social cognition test results in patients and the DEX-self, DEX-proxy, and DEX-dif scores are shown in Table 5. Concerning the DEX-proxy ratings, significant but weak correlations were found with the FEEST total score, FEEST Anger score, FEEST Disgust score, the Hayling score, and the BEES score (lower scores on these items were associated with more problems on the DEX-proxy scale). Thus, the worse the emotion recognition performance and the lower the scores on behaviour regulation and empathy in patients, the more behavioural problems were indicated by the proxy. The FEEST Anger score, the Faux-Pas empathy score, and the Hayling score correlated significantly with the DEX-dif score (lower scores on the FEEST Anger score, Faux-Pax empathy score, and Hayling score were associated with a lower DEX-dif score).

**Table 5:** Correlations between social cognition test results in patients and DEX-scores.

	DEX-self	DEX-proxy	DEX-dif (= self − proxy)
FEEST total score	-0.217**	-0.254*	0.072
FEEST-anger	-0.021	-0.225**	0.216**
FEEST-disgust	-0.268*	-0.277*	0.050
FEEST-fear	-0.155	-0.050	-0.086
FEEST-happiness	-0.067	-0.069	0.012
FEEST-sadness	-0.197**	-0.115	-0.056
FEEST-surprise	0.091	-0.016	0.098
Cartoons	-0.071	-0.137	0.079
Faux-Pas detection	-0.121	0.082	-0.192**
Faux-Pas empathy	0.092	-0.166	0.249*
Hayling	-0.087	-0.265*	0.195**
BEES	-0.091	-0.195**	0.121

FEEST = Facial Expression of Emotion: Stimuli and Tests; BEES = Balanced Emotional Empathy Scale; DEX = Dysexecutive Questionnaire

* p<0.01

** p<0.05

Table 6 shows the correlations between the social cognition test results and the four DEX subscale scores that were calculated from the DEX-proxy score. All significant correlation coefficients were negative, indicating that worse performances on the social cognition test measures were associated with more problems on the DEX subscales reported by the proxy.

**Table 6:** Correlations between social cognition test results in patients and DEX-proxy subscale scores.

	DEX-SC	DEX-BESR	DEX-EC	DEX-MC
FEEST total score	-0.192**	-0.233**	-0.242**	-0.244**
Cartoons	-0.125	-0.125	-0.135	-0.119
Faux-Pas detection	0.162	0.016	0.047	0.151
Faux-Pas empathy	-0.231**	-0.229**	-0.098	-0.141
Hayling	-0.202**	-0.263*	-0.309*	-0.156
BEES	-0.266*	-0.107	-0.188**	-0.194**

FEEST = Facial Expression of Emotion: Stimuli and Tests; BEES = Balanced Emotional Empathy Scale; DEX = Dysexecutive Questionnaire

* p<0.01.

** p<0.05.

## Discussion

Our study found a significant relationship between deficits in emotion recognition, empathy and behaviour regulation in stroke patients, and behavioural changes reported by significant others. This finding supports the hypothesis that deficits in aspects of social cognition may underlie behavioural deficits after stroke.

Stroke patients performed significantly worse than healthy controls on social cognition tests measuring emotion recognition, ToM and behaviour regulation in the long term after stroke. This is in line with previous studies.[6–10] It is interesting to find that social cognition impairments are still present at such a long time after stroke, even in a group of mildly affected stroke patients.

The majority of our patients (80.7%) suffered a minor stroke (NIHSS<5). Still, social cognition impairments were found in this relatively non-disabled patient population. It could, however, explain why we did not find differences between right- and left hemisphere stroke patients. Deficits in social cognition are associated with lesions in the right prefrontal cortex, the right superior temporal gyrus, and the temporo-parietal junction.[31] Generally, minor strokes do not affect these areas, as most of them are lacunar infarcts involving small penetrating arteries in the deep areas of the brain. In many studies comparing social cognition in right- and left hemisphere strokes, lacunar infarcts were excluded or comprised just a small amount of all strokes.[6] We also did not find differences between vertebrobasilar and anterior circulation stroke patients. One would expect that social cognition impairments are less common in vertebrobasilar circulation patients, but impairments are known to be present in this group as well. In recent literature, the association between vertebrobasilar stroke or other cerebellar diseases, and impaired emotion recognition was found.[32–34] Furthermore, functional MRI studies showed that the cerebellum is critically implicated in social cognition.[35,36]

It is up for discussion whether age and educational level should also have been included as covariates in the ANCOVA, because chi-square and t-tests on differences between patients and controls approached significance on these factors (age: t = -1.8, p = 0.08; and education level: X^2^ = 2.5, p = 0.12). Therefore, we also performed these analyses, and found that the results on the FEEST and Cartoon test did no longer differ between patients and controls (S1 Table). However, we feared that applying these ANCOVA’s may imply a form of overcorrection, which might even lead to a type II error. We deem it of clinical relevance to signal even mild impairments in social cognition in this group; not detecting effects that are there (type II error), prohibiting patients from getting the appropriate care, would in our opinion be more harmful for patients than the reverse (type I error). Hence, we presented the ANCOVA with sex as the only covariate, since this was the only demographic factor which differed significantly between patients and controls.

Mean DEX-scores were higher on the DEX-self rating version than on the DEX-proxy rating version, indicating that patients reported more problems about themselves than proxies did about them. As a consequence mean DEX-dif score was positive, which could be interpreted as normal self-awareness. However, lower DEX-dif scores correlated significantly with lower scores on the FEEST Anger, Faux-Pas empathy and Hayling. Hence, impaired self-awareness is likely to be related to deficits in social cognition, such as emotion recognition (anger), empathy and behaviour regulation. Although mean DEX-dif score was positive, 38.7% of patients had a negative DEX-dif score (i.e., these patients reported less problems than proxies did about them).

In the study of Spikman et al[14], mean DEX-self and DEX-proxy scores in their patient group with moderate to severe TBI were higher than in our present group of stroke patients. In TBI-patients (especially severe TBI), prefrontal brain damage is commonly found since prefrontal areas are specifically vulnerable to TBI. It is known that the presence of social behavioural problems is related to damage to inferior and medial prefrontal areas.[37,38] Generally, (minor) strokes do not affect these prefrontal areas. This might explain why TBI patients and their proxies may experience more behavioural problems, and thus, why mean DEX scores in TBI patients are higher than in our stroke patients. Nevertheless, even in stroke patients with a relatively favorable outcome (in terms of stroke severity), behavioural problems were found. This suggests that we should look beyond the location of brain injury by studying cerebral networks.[39] It is well known that stroke not only affects local connectivity but can also cause remote brain changes, as shown by functional MRI, Positron Emission Tomography (PET), and diffusion tensor imaging (DTI) studies.[40] Voxel-based lesion-symptom mapping results in patients with penetrating TBI showed that impairment in facial emotion recognition was due to damage in a bilateral fronto-temporo-limbic network, including medial prefrontal cortex, anterior cingulate cortex, left insula and temporal areas.[41] In patients who underwent resection of a low-grade glioma, lesion-symptom mapping showed that impairments in ToM were mainly related to the disruption of right fronto-parietal connectivity, and more specifically, to the degree of disconnection in the arcuate fasciculus and the cingulum.[42,43] Although lesion-symptom mapping studies on social cognition in stroke patients are lacking, it might be possible that even a minor stroke may interrupt a cerebral network, which is involved in social cognition processing.[39,44] Whether DTI-based measures of brain connectivity predict social cognition impairments as well is currently being investigated by the Prediction of Cognitive Recovery After Stroke (PROCRAS) investigators.[45]

The DEX is a broad measure of behavioural problems collectively known as the dysexecutive syndrome, that is, changes in emotion, personality, motivation, behaviour, executive functioning and cognition. The four subscales were designed to measure these different aspects separately. In relation to social cognition, we were most interested in the DEX-SC and DEX-BESR subscales, which measure social-emotional processes in behaviour. In addition to our overall finding that deficits on social cognition tasks were significantly, though weakly, related to behavioural problems in daily life, we found specific relationships between separate aspects of social cognition and separate categories of behavioural problems, represented by the different DEX subscales. Emotion recognition was related to all four subscales, but the Faux-Pas empathy (which is a reflection on feelings of a character in the Faux-Pas stories) only related to the two social-emotional subscales. The BEES, another indication of empathy, but measuring someone’s empathic reaction in hypothetical situations, was related to both the DEX-SC, the DEX-MC, and the DEX-EC subscales, but not to the DEX-BESR subscale. The Hayling, measuring behavioural regulation and inhibition, was in addition to the social-emotional subscales also related to the DEX-EC subscale, reflecting that the ability to stop behaviour is also important in executive task conditions. Correlations between the social cognition measures and the DEX subscales were not substantially different from the correlations with the total proxy score.

Just like emotional and cognitive problems, social cognitive and behavioural problems are invisible consequences of stroke. The invisibility of these consequences and the fact that stroke patients may experience impaired self-awareness, may lead to difficult situations in relationships and family. Relatives may feel at their wit’s end as they don’t understand the patients’ behaviour and eventually can’t handle the situation anymore. Now we know that there is a correlation between social cognition impairments and behavioural problems, the next step would be the identification of patients at risk of behavioural problems, so that targeted therapy can be given. Although only cross-sectional data were presented, and no statement can be made about causality, it is conceptually quite obvious that impairments in social information processing are underlying social behaviour, and not vice versa. Since social cognition tests can be performed in the acute stages after stroke, whereas behavioural problems only reveal themselves in the course of time, early detection of social cognition problems might contribute to the identification of patients at risk of behavioural problems. In this group of patients, focused psycho-education can be given to both patients and relatives. Also, when social cognition impairments are present, specific social cognitive treatment can be started, which has been proven effective in TBI patients.[46] Although this treatment may also be effective in stroke patients, this is a topic for further investigation.

One of the strengths of our study is that the most salient aspects of social cognition were assessed in a large sample of stroke patients. Moreover, we are the first to study the relationship between social cognition and behavioural changes after stroke, based on proxy ratings.

Some limitations of our study should be mentioned. First, no brain-imaging characteristics were assessed in our study. This could have told us more about lesion sites. Second, a disadvantage of examining social cognition in the long term after stroke is that only the most motivated patients may be willing to participate in extensive neuropsychological assessment. Nevertheless, no significant differences were found in stroke characteristics between the 119 patients left at T6 and the resigned patients. Another limitation is that we could not guarantee that all participating patients were free of pre-history personality problems that might have influenced performances on social cognition tests, or might have played a role in behavioural problems. Presence of psychiatric problems was not an exclusion criterion. Furthermore, the use of partners as healthy controls is a matter of debate, as they may not be naive to the purpose of the study, and their own behaviour may reflect or over-compensate social deficits in the patient they live with. Finally, since our study contains cross-sectional data, we could only examine the relationship between social cognition and behaviour in the long term. More research is needed to assess whether social cognition impairments in the acute stages after stroke can predict behavioural problems in the long term.

## Conclusions

Social cognition impairments are present in the long term after stroke, even in a group of mildly affected stroke patients. Most of these impairments also turned out to be associated with a broad range of behavioural problems as rated by proxies of the patients. Although only cross-sectional data were presented, this strengthens the proposal that social cognition impairments are part of the underlying mechanism of behavioural change. When patients at risk of behavioural problems could be identified in the early stages after stroke by performing social cognition tests, targeted social cognitive treatment can be given. Whether such treatment, that has been proven effective in TBI patients, is also effective in stroke patients, is a topic for further investigation.

## Supporting information

S1 Table Social cognition test results 3–4 years after stroke (ANCOVA with sex, age and educational level as covariates). (DOCX)

S1 Dataset SPSS data file. (SAV)
