# Supplementary material for: Social cognition impairments are associated with behavioural changes in the long term after stroke
Source: PLoS One. 2019 Mar 15;14(3):e0213725. doi: 10.1371/journal.pone.0213725 (PMC6420004; doi:10.1371/journal.pone.0213725)
Supplement: S1 Table — (DOCX) [file pone.0213725.s001.docx]

S1 table. Social cognition test results 3-4 years after stroke (ANCOVA with sex, age and educational level as covariates)

|  | Stroke patients (n=119) | Healthy controls (n=50) | ANCOVA | | |
| --- | --- | --- | --- | --- | --- |
| Test measures | Mean (SD) | Mean (SD) | F | p-value | Effect size |
| FEEST total score  FEEST-anger  FEEST-disgust  FEEST-fear  FEEST-happiness  FEEST-sadness  FEEST-surprise | 42.66 (6.2)  6.69 (2.2)  6.69 (2.4)  4.70 (2.2)  9.73 (0.6)  6.06 (2.0)  8.78 (1.5) | 45.02 (6.2)  7.79 (1.9)  7.27 (2.1)  4.81 (2.5)  9.79 (0.5)  6.50 (1.8)  8.85 (1.2) | 0.419  3.211  0.004  0.124  0.037  0.535  0.057 | 0.518  0.075  0.950  0.725  0.847  0.466  0.812 | 0.38  0.54  0.26  0.05  0.11  0.23  0.05 |
| Cartoon test | 21.04 (6.9) | 22.75 (5.9) | 0.636 | 0.427 | 0.27 |
| Faux Pas detection | 9.21 (1.0) | 9.12 (0.7) | 0.849 | 0.358 | -0.10 |
| Faux Pas empathy | 2.99 (1.2) | 3.28 (1.2) | 0.586 | 0.445 | 0.24 |
| Hayling | 3.02 (1.8) | 4.65 (1.4) | 23.977 | **<0.001** | 1.01 |
| BEES | 32.22 (21.5) | 35.60 (26.1) | 0.582 | 0.447 | 0.14 |

FEEST=Facial Expression of Emotion: Stimuli and Tests; BEES=Balanced Emotional Empathy Scale
